# Beyond Clear Margins: Oncologic Risk and Reconstructive Planning in Head and Neck Cutaneous Squamous Cell Carcinoma—A Structured Narrative Review

**DOI:** 10.3390/reports9030280

**Published:** 2026-08-23

**Authors:** Iris-Iuliana Adam, Liliana Vecerzan, Bogdan Moldovan, Raluca-Gabriela Miulescu, Alexandru-Petru Ciucu, Alina-Bianca Iacob, Alina Ormenișan

**Affiliations:** 1 ‘St. Constantin’ Hospital, 500299 Brașov, Romania; bogdan.moldovan@spitalulsfconstantin.ro (B.M.); miulescuraluca@yahoo.com (R.-G.M.); alina.iacob@umfst.ro (A.-B.I.); 2 Dental Elite Clinics, 500187 Brașov, Romania; alexandru.ciucu@dentalelite.ro; 3 Faculty of Medicine, “Lucian Blaga” University, 550169 Sibiu, Romania; 4 ‘Dr. Alexandru Augustin’ Military Hospital, 550024 Sibiu, Romania; 5 Emergency Clinical County Hospital Târgu Mureș, 540136 Târgu Mureș, Romania; alina.ormenisan@umfst.ro; 6 Faculty of Dental Medicine, George Emil Palade University of Medicine, Pharmacy, Science, and Technology of Târgu Mureș, 540142 Târgu Mureș, Romania

**Keywords:** cutaneous squamous cell carcinoma, head and neck, reconstruction, aesthetic outcome, patient-reported outcome, perineural invasion, immunosuppression, surgical margins, structured narrative review, research framework

## Abstract

Background: Head and neck cutaneous squamous cell carcinoma (HNcSCC) presents intersecting oncologic, functional, and reconstructive challenges. Although numerous clinicopathologic factors have been associated with recurrence, metastasis, and survival, their relationship with reconstructive complexity and patient-centered outcomes remains insufficiently studied. This review aimed to examine how established oncologic risk factors might inform reconstructive planning while distinguishing measured reconstructive evidence from hypothesis-generating clinical inferences. Methods: A structured PubMed/MEDLINE search conducted through 15 July 2026 was used to identify the literature addressing clinicopathologic prognostic factors in HNcSCC. Twenty-nine prognostic publications were retained for structured charting. Additional reconstructive, functional, aesthetic, and patient-reported outcome sources were identified through reference-list screening and were used solely for narrative contextualization. Because no dedicated multi-database systematic search of reconstructive outcomes was performed, the article is presented as a structured narrative review and hypothesis-generating research framework rather than a systematic review of reconstructive evidence. Results: The prognostic literature reported associations between adverse oncologic outcomes and factors including tumor size and depth, perineural invasion, lymphovascular invasion, poor differentiation, immunosuppression, recurrent disease, positive margins, nodal involvement, and extranodal extension. However, most of these studies did not measure post-excision defect characteristics, reconstructive technique, wound complications, functional recovery, scar quality, aesthetic outcomes, or patient-reported outcomes. Direct reconstructive evidence was limited and predominantly derived from site-specific, mixed-histology, technical, or methodological publications. Consequently, clinicopathologic factors should be regarded as potential upstream variables for future investigation rather than validated predictors of reconstructive outcomes. Conclusions: Current evidence supports oncologic risk stratification more strongly than prediction of reconstructive difficulty or patient-centered outcomes in HNcSCC. Prospective studies should jointly measure patient, tumor, treatment-field, defect, reconstructive, functional, aesthetic, and patient-reported variables. The proposed framework is intended to guide such research and is not a validated clinical prediction model.

## 1. Introduction

Cutaneous squamous cell carcinoma (cSCC) is the second most common non-melanoma skin cancer and represents a significant public health burden due to its continuously increasing incidence worldwide [1,2]. Although the majority of lesions are successfully treated with surgical excision, a subset of tumors exhibits aggressive biological behavior characterized by local recurrence, regional lymph node metastasis, distant dissemination, and disease-specific mortality [3]. Among all anatomical locations, the head and neck region accounts for approximately 80–90% of cSCC cases because of chronic ultraviolet radiation exposure, making head and neck cutaneous squamous cell carcinoma (HNcSCC) the most clinically relevant subtype [4,5].

For patients, oncologic clearance is only one component of the outcome, because facial surgery may leave lasting functional and aesthetic consequences. A clear margin may leave a defect that crosses facial aesthetic subunits, removes cartilage or bone, exposes the orbit or parotid bed, interrupts the facial nerve, or requires staged reconstruction. In the central face, a technically viable repair may still produce nasal obstruction, ectropion, or oral incompetence. Other visible consequences can include contour asymmetry, color mismatch, a conspicuous scar, or a prolonged sequence of revisions. Oncologic and reconstructive studies have largely evolved in parallel: one predicts recurrence and survival, whereas the other describes tissue replacement, reconstructive options, and aesthetic subunits [6,7,8,9,10,11,12,13,14,15].

This separation creates a clinically important blind spot. Tumor biology may influence the anticipated extent of resection, but the actual post-excision defect is a more immediate determinant of the reconstruction that follows. Diameter, depth, perineural or parotid involvement, previous treatment, and repeated margin clearance are plausible upstream variables; however, these pathways are rarely measured together with reconstructive outcomes in the same dataset [6,7,8,9,10,11,12,13].

Therefore, this review aimed to examine the conceptual interface between established oncologic risk factors and reconstructive planning in HNcSCC. Specifically, we sought to distinguish variables supported by direct reconstructive measurements from factors whose reconstructive relevance remains inferred, identify defect- and treatment-field characteristics that should be incorporated into future studies, and propose a hypothesis-generating framework for prospective validation. The review was not intended to establish validated predictors of reconstructive complications, functional impairment, or aesthetic outcomes.

## 2. Materials and Methods

### 2.1. Review Design and Scope

This article was developed as a structured narrative review and hypothesis-generating research framework. A structured literature search was used to identify publications addressing clinicopathologic prognostic factors in HNcSCC. Reconstructive, functional, aesthetic, and patient-reported outcome sources identified through reference-list screening were used for narrative contextualization and were not treated as a systematically assembled reconstructive dataset.

The review explicitly distinguished findings measured in the original publications from author-generated clinical hypotheses. Direct evidence required that a publication measured a defect characteristic, reconstructive technique, postoperative complication, functional outcome, aesthetic outcome, revision procedure, or patient-reported outcome. When an oncologic factor was discussed as potentially relevant to reconstruction without measurement of such an endpoint, it was classified as an indirect, hypothesis-generating association.

### 2.2. Scope and Source-Selection Framework

The clinical scope included adults with primary, recurrent, locally advanced, or nodal metastatic HNcSCC. Publications addressing cSCC from all anatomical sites were retained when they provided transferable information on high-risk clinicopathologic factors. Mixed-histology facial or scalp cohorts were used only for reconstructive context and were labeled accordingly when cSCC-specific data could not be separated. The scope and source-handling framework is summarized in Table 1. These criteria define the boundaries of the narrative review and should not be interpreted as eligibility criteria for a comprehensive systematic review of reconstructive interventions.

### 2.3. Literature Identification

A structured PubMed/MEDLINE search was conducted from database inception through 15 July 2026 to identify publications addressing clinicopathologic prognostic factors in HNcSCC. Eight keyword-based searches were performed separately, and the exact search phrases are reproduced verbatim in Supplementary Methods S1. The search was designed to identify oncologic prognostic literature and was not intended as a comprehensive systematic search of reconstructive outcomes.

After relevance assessment, 29 prognostic publications were retained for structured charting. Titles, abstracts, and available full texts were reviewed against the narrative scope. The process was not performed as independent duplicate screening, and no claim of exhaustive systematic coverage is made.

Additional publications addressing reconstruction, functional outcomes, aesthetic assessment, scar evaluation, and patient-reported outcomes were identified through backward reference-list screening. These sources were used only to provide narrative and methodological context. No claim is made that this contextual literature represents an exhaustive reconstructive evidence base.

### 2.4. Data Charting, Source Verification, and Evidence Classification

Charted domains included study design, sample size, population, clinical and histopathologic variables, measured outcomes, defect-level variables, reconstructive technique, complications, functional measures, aesthetic assessment, follow-up, and limitations. Only findings explicitly reported in the source publication were recorded as study-level results. Direct evidence required a measured defect, reconstructive, functional, complication, revision, aesthetic, or patient-reported endpoint. Potential reconstructive implications not measured by the source were classified separately as author-generated hypotheses and were not attributed to the original investigators.

### 2.5. Narrative Synthesis and Limitations of Appraisal

Because this article is a structured narrative review rather than a systematic review, no formal study-level risk-of-bias grading was undertaken. Study design, population, outcome measurement, confounding, generalizability, and potential cohort overlap were considered descriptively. Findings were not statistically pooled, and qualitative descriptors such as “strong” or “moderate-to-strong” were avoided because reproducible grading criteria were not available.

### 2.6. Use of Generative Artificial Intelligence (GenAI)

**Figure 1:**
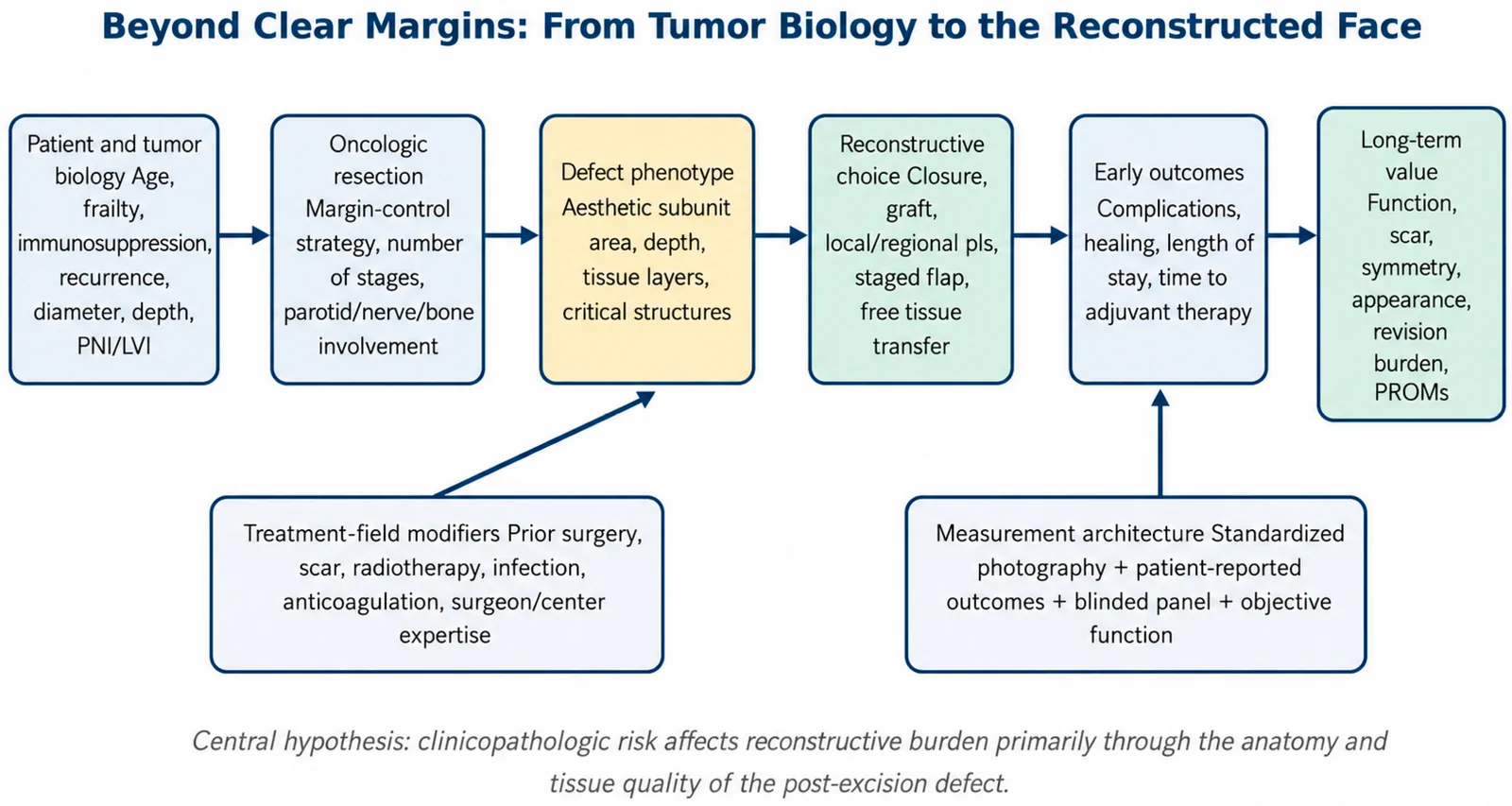
Hypothesis-generating conceptual pathway linking clinicopathologic factors, surgical resection, post-excision defect characteristics, reconstructive strategy, postoperative complications, functional recovery, aesthetic outcome, and patient-reported outcomes in HNcSCC. The arrows represent proposed relationships for future testing and do not indicate demonstrated causality or mediation.

ChatGPT (OpenAI, GPT-5.5) was used to support language editing, manuscript organization, and preliminary structuring of the conceptual framework and evidence tables. It was not treated as a source of scientific evidence. During revision, study-level statements and bibliographic records retained in the reconstructive table were checked against available source records and source text, and unsupported AI-assisted statements were removed. The authors remain responsible for the interpretation, conclusions, and final manuscript. The hypothesis-generating conceptual framework is presented in Figure 1.

## 3. Results

### 3.1. Characteristics of the Evidence Base

The structured prognostic literature set comprised 29 publications addressing clinicopathologic factors and oncologic outcomes in cSCC, with particular emphasis on head and neck disease (Table S1). These publications were used to describe patient-, tumor-, and nodal-level associations with recurrence, metastasis, or survival; they were not treated as evidence of reconstructive outcomes unless such outcomes were explicitly measured.

The prognostic literature was predominantly retrospective and observational, with additional prospective cohorts, systematic reviews, a clinician survey with a literature review, and a secondary analysis of a randomized trial. Sample sizes, populations, staging systems, outcome definitions, follow-up, and multivariable adjustment varied substantially. Several large multicenter publications may also contain overlapping source cohorts and were therefore not interpreted as fully independent replications.

The revised Supplementary Materials contain 42 unique publications: 29 prognostic publications in Table S1 and 13 distinct reconstructive, functional, aesthetic, technical, or methodological sources in Table S2, with no publication repeated across the two tables. The contextual sources were classified according to whether they provided direct clinical reconstructive outcomes, mixed-histology or site-specific evidence, technical guidance, or outcome-measurement methodology.

The predominant outcomes in the prognostic set were local recurrence, nodal or distant metastasis, disease-specific survival, overall survival, and staging performance. Post-excision defect dimensions, missing tissue layers, reconstructive technique, flap or graft failure, complications, function, scar quality, aesthetic assessment, patient satisfaction, and revision burden were generally not measured. Accordingly, the review identifies an evidence gap rather than validated clinicopathologic predictors of reconstructive difficulty or outcome.

No prognostic publication was counted as direct reconstructive evidence solely on the basis of biological plausibility. Table 2 therefore separates measured oncologic associations from direct reconstructive evidence and from author-generated hypotheses. Supporting references and evidence counts are reported for each domain.

### 3.2. Patient-Level Predictors: Age, Frailty, and Immunosuppression

Increasing age was associated with adverse tumor characteristics and reduced survival in portions of the prognostic literature, but chronological age alone should not be considered a contraindication to reconstruction. Its clinical significance is better interpreted alongside frailty, comorbidity, anesthesia tolerance, rehabilitation potential, and competing mortality. Local tissue laxity should be assessed independently of age: increased cervical skin laxity may facilitate local flap mobilization, whereas limited laxity—particularly on the scalp or in previously operated fields—may restrict primary closure and local flap advancement. These field characteristics were not measured in the prognostic studies and should be documented directly in future reconstructive cohorts.

Immunosuppression was associated with adverse oncologic outcomes across several prognostic publications. However, the retained prognostic studies did not establish that immunosuppression predicts infection, dehiscence, graft loss, flap complications, or delayed adjuvant therapy. These remain biologically plausible hypotheses that require direct prospective measurement rather than inference from recurrence or survival data.

### 3.3. Tumor-Level Predictors: Size, Depth, Differentiation, PNI, and LVI

Tumor diameter and invasion depth were repeatedly associated with oncologic progression. They may be investigated as upstream variables in reconstructive research, but the retained prognostic studies did not demonstrate a direct relationship with flap selection, complications, function, or aesthetics. Tumor dimensions cannot substitute for measurement of the actual post-excision defect, particularly after Mohs or staged margin-controlled surgery.

Perineural invasion illustrates the difference between oncologic and reconstructive evidence. Incidental microscopic PNI may influence adjuvant decisions, whereas clinically evident named-nerve spread may coincide with procedures involving the facial nerve, parotid, or skull base. However, the prognostic literature rarely reported nerve reconstruction, facial function, eye protection, static suspension, or patient-reported disability. Lymphovascular invasion likewise indicated aggressive biology but was not directly linked to reconstructive endpoints.

### 3.4. Margins, Recurrent Disease, and Treatment-Field Quality

Negative margins remain the primary oncologic endpoint. Repeated excision, delayed margin confirmation, recurrent disease, previous surgery, and previous radiotherapy may influence reconstructive planning, but their effects should be measured rather than assumed. Previous radiotherapy should not be treated as a binary variable: potential effects may vary with dose, field extent, tissue volume, fractionation, and interval since treatment. Future cohorts should record these parameters together with fibrosis, atrophy, telangiectasia, tissue mobility, recipient-vessel location, margin method, number of excision stages, timing of reconstruction, and time to adjuvant therapy.

### 3.5. Nodal and Parotid Disease

Nodal size, number of involved nodes, bilateral disease, extranodal extension, and parotid or cervical involvement were investigated primarily as oncologic variables. Parotidectomy and neck dissection nevertheless create a surgical context that differs substantially from a localized cutaneous defect and may involve vessel exposure, facial nerve dysfunction, contour loss, or soft-tissue volume loss. Future reconstructive studies should analyze this group separately and measure facial symmetry, dynamic and static facial function, neck contour, shoulder morbidity, and radiation-related change rather than infer these outcomes from nodal stage.

### 3.6. Direct Reconstructive and Aesthetic Evidence

The most informative direct aesthetic evidence came from nasal reconstruction studies rather than cSCC-specific cohorts. Mureau and colleagues assessed functional and aesthetic outcomes after nasal reconstruction [6]. Moolenburgh and colleagues evaluated patient, professional-panel, and lay-observer perspectives [7,8], demonstrating that these perspectives should be reported separately rather than treated as interchangeable measures.

Technical sources describe the aesthetic subunit principle and the restoration of nasal lining, structural support, and skin cover for complex nasal defects [9,10]. Scalp and calvarial literature describes technique selection according to defect dimensions, depth, exposed bone or dura, infection, previous irradiation, and tissue quality [11,12,13,44,45,46]. These principles are anatomically relevant, but many sources are reviews, technical frameworks, or mixed-histology cohorts rather than HNcSCC-specific comparative studies. Table 3 therefore identifies measured endpoints, evidence counts, and domains in which no direct evidence was identified.

## 4. Discussion

### 4.1. Main Findings and Interpretation

The central finding of this review is an asymmetry between oncologic prognostication and reconstructive outcome research in HNcSCC. Tumor diameter, invasion depth, differentiation, PNI, LVI, immunosuppression, recurrence, margin status, nodal burden, and extranodal extension have been investigated mainly in relation to recurrence, metastasis, or survival [16,17,18,19,20,21,22,23,24,25,26,27,28,29,30,31,32,33,34,35,36,37,38,39,40,41,42,43,47,48,49,50,51,52]. The same literature rarely records post-excision defect anatomy, reconstructive tier, number of procedures, functional recovery, scar quality, or patient-rated appearance. These variables should therefore not be described as validated predictors of reconstructive outcomes.

Oncologic risk and reconstructive burden are related but not interchangeable. We propose the post-excision defect as a conceptual intermediate variable between disease, surgical resection, and reconstructive outcome. This relationship has not been demonstrated through mediation analysis or prospective longitudinal data. The framework should therefore be interpreted as a hypothesis for future testing, with defect anatomy placed at the center of measurement rather than assumed from tumor stage alone.

### 4.2. Tumor Biology as an Upstream Determinant of Reconstructive Burden

Tumor size and depth are established oncologic variables [16,17,18], but their contribution to reconstructive burden remains unquantified. They should be evaluated as potential upstream indicators rather than assumed surrogates for the definitive defect. Margin-controlled excision may preserve tissue in one patient, whereas ill-defined borders, recurrent growth, or repeated re-excision may enlarge the defect in another patient with a tumor of similar clinical size. Future studies should record both preoperative tumor dimensions and postoperative defect dimensions.

PNI illustrates this distinction. Incidental microscopic PNI and clinically evident spread along a named nerve occupy different positions on the oncologic spectrum and may be associated with different surgical fields. Existing studies identify PNI as an adverse oncologic factor [16,17,18,25,26,27,29,31,32,47,48], but they seldom report facial function, corneal protection, sensory loss, nerve reconstruction, rehabilitation, or patient-reported disability. LVI and poor differentiation likewise signal aggressive biology, yet their reconstructive effects remain unmeasured. They are candidate variables for prospective research, not validated reconstructive predictors.

### 4.3. Patient Factors, Recurrence, and Treatment-Field Quality

Patient-level variables modify both the feasibility and the consequences of reconstruction. Increasing age is associated with adverse tumor characteristics and poorer survival in many HNcSCC cohorts, but chronological age alone is an inadequate measure of operative vulnerability. Frailty, cardiopulmonary reserve, nutritional status, diabetes, smoking, anticoagulation, functional independence, and rehabilitation potential are more likely to determine whether a prolonged microvascular procedure, a staged reconstruction, or a simpler wound strategy is appropriate. Thus, age should be interpreted as contextual information rather than as an isolated contraindication to complex reconstruction.

Immunosuppression deserves particular attention because it may influence the reconstructive pathway through two mechanisms. First, transplant recipients and patients with hematologic or treatment-related immunosuppression are more likely to develop aggressive, recurrent, and multifocal cSCC. Second, impaired immune function may plausibly increase infection, dehiscence, graft loss, delayed wound healing, and interruption of adjuvant therapy. The first pathway is well supported by oncologic evidence, whereas the second remains insufficiently quantified in HNcSCC-specific reconstructive cohorts. This distinction should be preserved in clinical interpretation: a biologically credible association is not equivalent to a measured reconstructive risk.

Recurrent tumors and previously treated fields may present scars, distorted tissue planes, reduced mobility, prior grafts or flaps, and radiation-related tissue changes. The magnitude of radiation effects is likely to depend on dose, field, fractionation, tissue volume, and time since treatment; these details were not captured by the prognostic literature. Current European guidance maintains complete oncologic excision as the primary objective [3,53]. Future reconstructive studies should additionally record margin assessment method, number of excision stages, previous surgery, radiotherapy parameters, clinically observed tissue quality, and the interval to adjuvant treatment.

### 4.4. Nodal and Parotid Disease as a Distinct Reconstructive Phenotype

Nodal metastasis and parotid or cervical involvement should be distinguished from localized cutaneous defects. Parotidectomy and neck dissection may involve facial nerve dysfunction, contour loss, exposure of major vessels, loss of soft-tissue volume, shoulder morbidity, or a larger adjuvant-treatment field. The staging literature provides prognostic information on nodal burden and extranodal extension [29,41,43], but reconstructive outcomes are rarely stratified by these variables. Future analyses should therefore separate localized resections from composite parotid, neck, orbital, or skull-base procedures.

### 4.5. Aesthetic and Functional Outcomes Require Multidimensional Assessment

The contextual reconstructive literature indicates that technical success, patient satisfaction, and observer-rated aesthetics are distinct outcomes. Nasal reconstruction studies reported differences between patient, professional-panel, and lay-observer assessments [6,7,8]. These perspectives should be reported separately. A global label such as “good cosmetic result” does not specify the measurement method and is therefore inadequate for reproducible outcome assessment.

The aesthetic subunit principle and replacement of missing lining, structural support, and skin cover are established technical concepts in nasal reconstruction [9,10]. Scalp and calvarial sources describe reconstruction according to defect size, depth, exposed bone or dura, surrounding tissue quality, and previous treatment [11,12,13,44,45,46]. These frameworks support defect-based planning, but much of the evidence is derived from mixed-histology, site-specific, retrospective, or technical sources. Anatomical transferability does not establish prognostic validity in HNcSCC.

Future studies should combine standardized clinical photography, blinded observer assessment, site-specific functional measures, complication reporting, revision burden, and patient-reported outcomes. The Nasal Obstruction Symptom Evaluation scale measures patient-perceived nasal obstruction [14], while the Patient and Observer Scar Assessment Scale permits parallel assessment of scar quality by patients and observers [15]. These outcomes should be reported separately rather than collapsed into a single success score.

### 4.6. Clinical Implications and a Hypothesis-Generating Research Framework

The proposed framework is intended solely to guide future research and should not be interpreted as a clinically applicable prediction model. No variables have been weighted, no scoring system or decision threshold has been defined, and predictive performance, calibration, and clinical utility have not been evaluated. Its purpose is to promote earlier multidisciplinary documentation of anticipated tissue loss, potential nerve or structural sacrifice, local tissue availability, staged reconstruction, surveillance needs, and compatibility with adjuvant treatment without compromising oncologic clearance.

Future research could evaluate two linked models rather than assume a single score. A preoperative model might examine frailty, immunosuppression, recurrence, tumor size, depth, subsite, clinical PNI, anticipated parotid or bone involvement, previous surgery, radiotherapy parameters, and local tissue mobility. A postoperative model could examine surface area, dimensions, depth, absent tissue layers, aesthetic subunits, exposed structures, margin status, and field quality in relation to technique, complications, revision, function, and aesthetics. Any model should be developed according to TRIPOD principles, assessed with PROBAST, and subjected to external validation and calibration before clinical use [54,55].

### 4.7. Methodological Strengths, Limitations, and Research Priorities

The primary contribution of this review is its explicit separation of measured reconstructive evidence from oncologic associations and author-generated hypotheses. This distinction reduces the risk of treating recurrence or survival factors as established predictors of flap failure, function, scar quality, or patient satisfaction. The framework is intended to make missing outcomes visible and to guide future study design.

Several limitations temper the conclusions. This was a single-database, structured narrative review rather than an exhaustive systematic search, and reconstructive sources were identified through reference-list screening. Screening and charting were not performed independently in duplicate; formal study-level risk-of-bias grading was not undertaken; and effect estimates were not quantitatively synthesized. The prognostic literature was predominantly retrospective, heterogeneous, and potentially affected by overlapping multicenter cohorts. Contextual reconstructive publications frequently included mixed histologies, selected tertiary-center populations, or technical reviews. Consequently, the proposed relationships are hypothesis-generating and should not be interpreted as validated causal or predictive effects.

The next step should be a prospective, multicenter HNcSCC reconstruction cohort with consecutive enrollment and a predefined minimum dataset. Patient variables should include frailty, comorbidity, immunosuppression, smoking, diabetes, nutritional status, and previous treatment; tumor variables should include size, depth, differentiation, PNI, LVI, recurrence, margins, nodal burden, and extranodal extension; defect variables should document dimensions, depth, aesthetic subunits, missing layers, and critical structures; and reconstructive variables should capture technique, operative stages, complications, reoperation, and time to adjuvant treatment. Standardized photographs, functional measures, scar assessment, patient satisfaction, and revision burden should be collected longitudinally. Only by measuring these domains in the same patient can the field determine whether tumor biology predicts the reconstructed face directly, indirectly through defect anatomy, or not at all.

## 5. Conclusions

Current HNcSCC literature supports oncologic risk stratification but does not validate clinicopathologic predictors of reconstructive difficulty, complications, functional impairment, aesthetic outcome, or patient experience. Most prognostic studies did not measure post-excision defect characteristics or reconstructive endpoints, while the available direct reconstructive literature was limited, site-specific, and frequently derived from mixed-histology or technical sources.

The post-excision defect is proposed as a conceptual intermediate variable for future investigation rather than as a demonstrated mediating phenotype. Prospective studies should jointly record standardized patient, tumor, treatment-field, defect, reconstructive, functional, aesthetic, and patient-reported variables. The framework presented here requires prospective development and external validation before any clinical application.

## Figures and Tables

**Table 1:** Scope and source-handling framework used in the structured narrative review.

Domain	Narrative Scope	Source Handling
Population	Adults with primary, recurrent, locally advanced, or nodal metastatic cSCC/HNcSCC.	Mucosal HNSCC excluded from the clinical scope; mixed skin-cancer cohorts used only for reconstructive context unless cSCC data were separable.
Predictors	Age/frailty, immunosuppression, tumor size and depth, ulceration, differentiation, PNI, LVI, margins, recurrence, nodal burden, ENE, and treatment history.	Molecular studies without clinical or reconstructive relevance were not charted.
Reconstruction	Primary closure, secondary intention, grafts, local or regional flaps, staged pedicled flaps, free tissue transfer, and structural grafting.	Technical and mixed-histology sources were used for anatomical or methodological context, not comparative HNcSCC inference.
Outcomes	Defect dimensions and layers, technique, complications, reoperation, function, scar, aesthetics, satisfaction, quality of life, and PROMs.	Undefined global cosmetic outcomes without a stated measurement method were treated as non-reproducible.
Design	Prognostic cohorts, validation studies, systematic reviews, reconstructive cohorts, and selected technical or measurement sources.	Evidence type and HNcSCC applicability were labeled explicitly; no source was assigned an unmeasured reconstructive finding.

**Table 2:** Summary of evidence domains and their reconstructive interpretation in HNcSCC.

Domain	Measured Findings	Reconstructive Interpretation	Evidence Category and Supporting Sources
Tumor size and depth	Larger diameter, thickness, depth, and extension beyond subcutaneous fat were studied primarily in relation to progression or metastasis.	Potential upstream variables for future defect-based research; not validated predictors of reconstructive technique or outcome.	Oncologic associations measured; reconstructive outcomes not measured in the prognostic set (representative sources: refs. [16,17,18,19,20,21,22,23,24,25,26]).
Differentiation, PNI, and LVI	These histopathologic features were associated with adverse oncologic outcomes in several cohorts.	May coincide with wider or composite surgery in selected clinical settings, but the relationship with reconstruction requires direct measurement.	Oncologic associations measured; direct reconstructive prediction not established (refs. [19,21,25,26,27,28,29,30,31,32]).
Immunosuppression	Transplant status, hematologic disease, and other immunosuppression were associated with recurrence, metastasis, or survival outcomes.	Infection, dehiscence, graft loss, and flap complications are hypotheses for prospective study, not findings from the prognostic sources.	Oncologic associations measured; reconstructive complications not directly measured (refs. [19,22,29,32,33,34,35,36,37]).
Margins and recurrence	Margin status and recurrent disease were evaluated mainly as oncologic variables.	Number of excision stages, prior operations, field quality, and timing of closure should be recorded directly.	Oncologic associations measured; reconstructive effect remains indirect (refs. [22,27,32,33,34]).
Nodal, parotid disease, and ENE	Nodal burden and ENE were investigated as prognostic variables in metastatic cohorts.	Composite parotid/neck procedures should be analyzed separately from localized cutaneous defects.	Oncologic associations measured; reconstructive outcomes rarely stratified (refs. [29,31,35,37,38,39,40,41,42,43]).
Defect anatomy and reconstructive technique	Defect dimensions, absent layers, exposed structures, and tissue quality were addressed in site-specific and mixed-histology reconstructive sources.	These are direct reconstructive variables and should be prospectively documented in HNcSCC cohorts.	Direct but predominantly mixed-histology/site-specific evidence (8 sources: refs. [9,10,11,12,13,44,45,46]).
Aesthetic, functional, and patient-reported outcomes	Nasal and scalp studies used patient reports, observer panels, functional assessment, standardized photography, or aesthetic scales.	Patient, observer, functional, scar, and revision outcomes should be reported separately.	Direct, site-specific or methodological evidence (6 sources: refs. [6,7,8,14,15,44]).

**Table 3:** Evidence-gap matrix linking clinicopathologic and defect-level variables with reconstructive, functional, aesthetic, and patient-reported outcomes.

Predictor/Domain	Oncologic Prognosis	Defect Extent	Reconstructive Complexity	Complications	Function	Aesthetic Outcome
Tumor diameter/depth	Measured in prognostic studies (multiple sources; refs. [16,17,18,19,20,21,22,23,24,25,26])	No direct evidence identified (*n* = 0)	No direct evidence identified (*n* = 0)	No direct evidence identified (*n* = 0)	No direct evidence identified (*n* = 0)	No direct evidence identified (*n* = 0)
Recurrence/prior surgery	Measured in prognostic studies (refs. [22,27,32,33,34])	No direct evidence identified (*n* = 0)	No direct evidence identified (*n* = 0)	No direct evidence identified (*n* = 0)	No direct evidence identified (*n* = 0)	No direct evidence identified (*n* = 0)
Perineural invasion	Measured in prognostic studies (refs. [16,17,18,25,26,27,29,31,32,47,48])	No direct evidence identified (*n* = 0)	No direct evidence identified (*n* = 0)	No direct evidence identified (*n* = 0)	No direct evidence identified (*n* = 0)	No direct evidence identified (*n* = 0)
Immunosuppression/transplant	Measured in prognostic studies (refs. [19,22,29,32,33,34,35,36,37])	No direct evidence identified (*n* = 0)	No direct evidence identified (*n* = 0)	No direct evidence identified (*n* = 0)	No direct evidence identified (*n* = 0)	No direct evidence identified (*n* = 0)
Margin status/re-excision	Measured in oncologic studies (refs. [27,33,34])	No direct evidence identified (*n* = 0)	No direct evidence identified (*n* = 0)	No direct evidence identified (*n* = 0)	No direct evidence identified (*n* = 0)	No direct evidence identified (*n* = 0)
Nodal/parotid disease and ENE	Measured in metastatic prognostic cohorts (refs. [29,31,35,37,38,39,40,41,42,43])	No direct evidence identified (*n* = 0)	No direct evidence identified (*n* = 0)	No direct evidence identified (*n* = 0)	No direct evidence identified (*n* = 0)	No direct evidence identified (*n* = 0)
Defect area and tissue layers	Not a primary prognostic endpoint	Direct, mixed-histology/site-specific evidence (*n* = 8; refs. [9,10,11,12,13,44,45,46])	Direct, mixed-histology/site-specific evidence (*n* = 8; refs. [9,10,11,12,13,44,45,46])	Direct evidence in selected cohorts (refs. [44,45,46])	Direct evidence in selected sources (refs. [6,44])	Direct evidence in selected sources (refs. [6,7,8,44])
Aesthetic subunit/tissue quality	Not a primary prognostic endpoint	Direct technical evidence (refs. [9,10,11,12,13])	Direct technical evidence (refs. [9,10,11,12,13])	Limited direct comparative evidence	Direct site-specific evidence (refs. [6,9,10])	Direct site-specific evidence (refs. [6,7,8,9,10,44])
Reconstructive technique	Not a primary prognostic endpoint	Direct reconstructive evidence (refs. [9,10,11,12,13,44,45,46])	Direct reconstructive evidence (refs. [9,10,11,12,13,44,45,46])	Direct cohort evidence (refs. [44,45,46])	Direct but heterogeneous evidence (refs. [6,9,10,11,12,13,44,45,46])	Direct but heterogeneous evidence (refs. [6,7,8,9,10,11,12,13,44,45,46])
PROM/panel-rated aesthetics	Not a primary prognostic endpoint	Not applicable	No direct prediction evidence identified	Not applicable	Direct methodological evidence (ref. [14])	Direct site-specific/methodological evidence (*n* = 5; refs. [6,7,8,15,44])

Evidence classification: “Direct” indicates that the specified endpoint was measured in the cited source. “No direct evidence identified” indicates that none of the retained sources measured the relationship. Oncologic associations were not reclassified as reconstructive findings. Counts refer to distinct contextual sources, not repeated prognostic publications.

## Data Availability

The search phrases, structured study-charting tables, and evidence classifications created for this review are provided in the Supplementary Materials. No individual participant data were generated.

## Supplementary Materials

The following supporting information can be downloaded at https://www.mdpi.com/article/10.3390/reports9030280/s1, Supplementary Method S1: Exact PubMed/MEDLINE keyword search phrases used to identify oncologic prognostic literature; Supplementary Table S1: structured charting of prognostic publications (*n* = 29); Supplementary Table S2: distinct contextual reconstructive, functional, aesthetic, technical, and methodological sources (*n* = 13).

## Abbreviations

The following abbreviations are used in this manuscript:

AJCC	American Joint Committee on Cancer
AJCC8	American Joint Committee on Cancer, 8th Edition
BWH	Brigham and Women’s Hospital
CC BY	Creative Commons Attribution License
cSCC	Cutaneous squamous cell carcinoma
DFS	Disease-free survival
DMR	Distant metastatic recurrence
DSS	Disease-specific survival
ENE	Extranodal extension
EADO	European Association of Dermato-Oncology
EADV	European Academy of Dermatology and Venereology
EDF	European Dermatology Forum
EORTC	European Organisation for Research and Treatment of Cancer
ESTRO	European Society for Radiotherapy and Oncology
ECS	Extracapsular spread
GenAI	Generative Artificial Intelligence
HNcSCC	Head and neck cutaneous squamous cell carcinoma
HNSCC	Head and neck squamous cell carcinoma
ITEM	Item-based nodal staging system
LVI	Lymphovascular invasion
MRI	Magnetic resonance imaging
NOSE	Nasal Obstruction Symptom Evaluation
OS	Overall survival
PET	Positron emission tomography
PMID	PubMed Identifier
PNI	Perineural invasion
PORT	Postoperative radiotherapy
POSAS	Patient and Observer Scar Assessment Scale
PRO	Patient-reported outcome
PROBAST	Prediction model Risk Of Bias ASsessment Tool
PROM	Patient-reported outcome measure
PROMs	Patient-reported outcome measures
RCT	Randomized controlled trial
SCC	Squamous cell carcinoma
S-ITM	Satellitosis/In-transit metastasis
TNM	Tumor–Node–Metastasis
TRIPOD	Transparent Reporting of a multivariable prediction model for Individual Prognosis Or Diagnosis
UEMS	European Union of Medical Specialists
