# Supplementary material for: Beyond Clear Margins: Oncologic Risk and Reconstructive Planning in Head and Neck Cutaneous Squamous Cell Carcinoma—A Structured Narrative Review
Source: Reports (MDPI). 2026 Aug 23;9(3):280. doi: 10.3390/reports9030280 (PMC13611058; doi:10.3390/reports9030280)
Supplement: Supplementary file 1 [file reports-09-00280-s001.zip › reports-4490254-supplementary.pdf]

Supplementary Methods S1. Structured PubMed/MEDLINE search phrases for oncologic prognostic literature

The following eight PubMed/MEDLINE keyword searches were performed to identify literature on clinicopathologic prognostic factors in HNcSCC. The search was not a dedicated systematic search of reconstructive interventions or outcomes.

Exact search phrases:

- cutaneous squamous cell carcinoma head and neck prognostic factors
- cutaneous squamous cell carcinoma head and neck histopathological predictors
- cutaneous squamous cell carcinoma perineural invasion head and neck
- cutaneous squamous cell carcinoma tumor thickness prognosis
- cutaneous squamous cell carcinoma differentiation prognosis
- cutaneous squamous cell carcinoma lymphovascular invasion
- cutaneous squamous cell carcinoma recurrence predictors
- high risk cutaneous squamous cell carcinoma head and neck

Table S1: Overview of All Studies included in the Review

No.	Study ID (Author, Year)	Study Design	N	Clinical Predictor	Histopathological Predictor	Outcome	Reported study-level findings	Oncologic interpretation	Limitations / reconstructive relevance
1	Lubov et al.; 2021	Systematic review	8535	Age, immunosuppression	Tumor depth, margins, lymph nodes	Metastasis	Immunosuppression and tumor depth were among the strongest predictors	High heterogeneity and predominance of retrospective studies limited direct comparisons.	Immunosuppression and tumor depth were among the strongest predictors of metastasis in HNcSCC. No direct reconstructive, functional, complication, or aesthetic endpoint was attributed unless explicitly reported.

No.	Study ID (Author, Year)	Study Design	N	Clinical Predictor	Histopathological Predictor	Outcome	Reported study-level findings	Oncologic interpretation	Limitations / reconstructive relevance
**2**	Watts et al.; 2021	International survey + literature review	105 specialists (survey)	Immunosuppression	Not evaluated	Prognostic stratification / AJCC staging	Current AJCC nodal staging shows limited prognostic performance in HNcSCC. Immunosuppression and soft tissue metastases were identified by clinicians as important factors that should be considered in prognostic stratification.	The findings support the need for a HNcSCC-specific staging system that incorporates additional clinically relevant prognostic factors beyond the current AJCC classification.	Based primarily on expert opinion and a limited body of published evidence, without direct patient-level outcome analysis. No direct reconstructive, functional, complication, or aesthetic endpoint was attributed unless explicitly reported.
**3**	Leus et al.; 2021	Retrospective cohort study	672	Age, tumor diameter	Tumor differentiation, invasion depth	Disease progression (local recurrence, nodal metastasis, distant metastasis)	In elderly patients, tumor diameter ≥40 mm, moderate differentiation, and invasion depth ≥2 mm were independent predictors of disease progression. Older patients also presented with larger tumors, poorer differentiation, and deeper invasion.	Age influences both tumor characteristics and prognostic factors, suggesting that age-specific risk stratification may improve management of head and neck cSCC.	Retrospective single-center study; findings require external validation in larger prospective cohorts. No direct reconstructive, functional, complication, or aesthetic endpoint was attributed unless explicitly reported.
**4**	Schachtel et al.; 2021	Retrospective cohort study	73	Recurrent disease, VII zonal extent, concurrent trigeminal nerve involvement	Positive surgical margins, pathological nodal disease	Disease-free survival, disease-specific survival, overall survival	Pathological nodal disease, positive surgical margins, greater facial nerve involvement, and concurrent trigeminal nerve spread were associated with significantly poorer survival outcomes.	Early diagnosis, complete surgical excision with negative margins, and postoperative radiotherapy improve outcomes in patients with facial nerve PNS from head and neck cSCC.	Retrospective single-center study with a relatively small sample size and inclusion limited to patients with facial nerve PNS. No direct reconstructive, functional, complication, or aesthetic endpoint was attributed unless explicitly reported.

No.	Study ID (Author, Year)	Study Design	N	Clinical Predictor	Histopathological Predictor	Outcome	Reported study-level findings	Oncologic interpretation	Limitations / reconstructive relevance
**5**	Bobin et al.; 2017	Retrospective cohort study	35	Age, immunosuppression, macroscopic facial nerve involvement, cervical lymph node metastases	Positive surgical margins	Overall survival, disease-specific survival in patients with parotid metastasis	Immunosuppression, older age, positive surgical margins, facial nerve involvement, and cervical lymph node metastases were independent predictors of poorer survival.	Early detection and complete excision of the primary tumor with negative margins may reduce the risk of parotid metastasis and improve patient outcomes.	Retrospective single-center study with a small sample size, limited to patients with parotid metastases. No direct reconstructive, functional, complication, or aesthetic endpoint was attributed unless explicitly reported.
**6**	Bachar et al.; 2016	Retrospective cohort study	71	Age, site of nodal metastasis	Tumor differentiation	Disease-free survival (DFS), disease-specific survival (DSS), overall survival (OS)	Poor tumor differentiation was independently associated with worse disease-free survival, while older age independently predicted poorer overall survival. The site of nodal metastasis was not associated with prognosis.	Age and tumor differentiation are important prognostic factors in metastatic head and neck cSCC, whereas the location of nodal metastasis appears to have limited prognostic value.	Retrospective single-center study with a relatively small cohort restricted to patients with metastatic disease. No direct reconstructive, functional, complication, or aesthetic endpoint was attributed unless explicitly reported.
**7**	Melo et al., 2025	Retrospective single-center cohort study	156	Transplant status, immunosuppression, advanced AJCC stage (III–IV)	Positive surgical margins, cervical lymph node metastasis with extranodal extension	Recurrence, overall survival, mortality	Transplant recipients and patients with cervical lymph node metastasis with extranodal extension had significantly higher recurrence rates. Positive surgical margins and extranodal extension were independent predictors of mortality.	Immunosuppression and transplant status are associated with poorer outcomes in locally advanced HNcSCC, highlighting the need for tailored risk stratification and management.	Retrospective single-center study focused exclusively on locally advanced tumors, which may limit generalizability. No direct reconstructive, functional, complication, or aesthetic endpoint was attributed unless explicitly reported.

No.	Study ID (Author, Year)	Study Design	N	Clinical Predictor	Histopathological Predictor	Outcome	Reported study-level findings	Oncologic interpretation	Limitations / reconstructive relevance
**8**	McLean et al.; 2012	Retrospective cohort study	95	Immunosuppression	Nodal extracapsular spread (ECS)	Overall survival (OS), disease-specific survival (DSS)	Immunosuppression and nodal extracapsular spread were independent predictors of poorer overall and disease-specific survival. The characteristics of the primary tumor did not significantly influence survival.	Immunosuppression and extracapsular spread are major adverse prognostic factors in patients with concurrent primary and metastatic HNcSCC.	Retrospective study restricted to patients with concurrent primary and nodal metastatic disease, limiting generalizability to all HNcSCC patients. No direct reconstructive, functional, complication, or aesthetic endpoint was attributed unless explicitly reported.
**9**	Jambusaria-Pahlajani et al.; 2025	Multicenter retrospective cohort study	23,166 localized cSCC tumors	Multiple clinical risk factors included in the riSCC model	Multiple pathological risk factors included in the riSCC model	Local recurrence, nodal metastasis, disease-specific death	The riSCC model demonstrated better prognostic performance than AJCC 8th edition and Brigham and Women's Hospital staging systems across all evaluated outcomes.	The riSCC model provides more accurate individualized risk stratification for patients with localized cSCC than currently available staging systems.	Retrospective study design; not restricted to head and neck cSCC. No direct reconstructive, functional, complication, or aesthetic endpoint was attributed unless explicitly reported.
**10 ***	Pahalyants et al.; 2025	Multicenter retrospective cohort study	8901	Age, immunosuppression, advanced BWH stage	Lymphovascular invasion	Satellitosis/in-transit metastasis (S-ITM), local recurrence, nodal metastasis, distant metastasis, disease-specific death	Older age, immunosuppression, advanced BWH stage, and lymphovascular invasion were independent risk factors for S-ITM. S-ITM was associated with significantly increased risks of recurrence, metastasis, and disease-specific death.	S-ITM represents an independent marker of aggressive disease and poor prognosis in patients with cSCC.	Retrospective multicenter design; the low incidence of S-ITM may have limited statistical power. No direct reconstructive, functional, complication, or aesthetic endpoint was attributed unless explicitly reported.

No.	Study ID (Author, Year)	Study Design	N	Clinical Predictor	Histopathological Predictor	Outcome	Reported study-level findings	Oncologic interpretation	Limitations / reconstructive relevance
**11**	Porceddu et al.; 2025	Secondary analysis of a prospective randomized phase III clinical trial	310	Nodal size ≥22 mm	Extranodal extension (ENE)	Disease-free survival (DFS), Overall survival (OS)	Patients with extranodal extension and nodal size ≥22 mm had significantly worse disease-free and overall survival, defining a distinct high-risk subgroup after postoperative radiotherapy.	Extranodal extension combined with nodal size ≥22 mm identifies patients with particularly poor prognosis who may benefit from intensified adjuvant treatment strategies.	Secondary analysis of a clinical trial; findings apply primarily to patients with high-risk nodal disease treated with postoperative radiotherapy. No direct reconstructive, functional, complication, or aesthetic endpoint was attributed unless explicitly reported.
**12**	Klein et al.; 2025	Multicenter retrospective cohort study	11930 patients (18760 tumors)	Immunosuppression, organ transplantation, chronic lymphocytic leukemia	High-risk tumor features (evaluated as covariates)	Local recurrence, distant metastasis, disease-specific death, major poor outcomes	Immunosuppression was an independent predictor of local recurrence, distant metastasis, disease-specific death, and major poor outcomes. Organ transplantation and chronic lymphocytic leukemia were associated with particularly poor prognosis.	Immunosuppression is an independent predictor of adverse outcomes in cSCC and should be incorporated into future risk prediction models.	Retrospective multicenter design with potential data heterogeneity; not restricted to head and neck cSCC. No direct reconstructive, functional, complication, or aesthetic endpoint was attributed unless explicitly reported.
**13**	Ebrahimi et al.; 2024	Multicenter retrospective cohort study	1151	Immunosuppression, nodal size, number of nodal metastases	Perineural invasion, extranodal extension	Disease-specific survival, overall survival, locoregional control	Immunosuppression, perineural invasion, extranodal extension, larger nodal size, and a higher number of metastatic lymph nodes were independently associated with worse survival and locoregional control.	Multiple clinicopathological factors provide more accurate prognostic stratification than AJCC8 staging alone, suggesting that the current staging system overemphasizes extranodal extension.	Retrospective design; included only patients with nodal metastatic HNcSCC treated with curative intent. No direct reconstructive, functional, complication, or aesthetic endpoint was attributed unless explicitly reported.

No.	Study ID (Author, Year)	Study Design	N	Clinical Predictor	Histopathological Predictor	Outcome	Reported study-level findings	Oncologic interpretation	Limitations / reconstructive relevance
**14**	Silberstein et al.; 2015	Retrospective cohort study	572 patients (725 tumors)	Tumor stage (T1 vs. T2)	Not specifically evaluated	Cervical lymph node metastasis	Cervical lymph node metastasis occurred in 1.3% of patients. T2 tumors had a significantly higher risk of nodal metastasis than T1 tumors, while no distant metastases were observed during follow-up.	Tumor stage is associated with the risk of cervical lymph node metastasis. Because the overall incidence of nodal metastasis is low, routine sentinel lymph node biopsy is not recommended for clinically node-negative patients.	Retrospective single-center study with a small number of metastatic events, limiting the evaluation of additional prognostic factors. No direct reconstructive, functional, complication, or aesthetic endpoint was attributed unless explicitly reported.
**15**	Luk et al.; 2021	Multicenter retrospective cohort study	1146	AJCC8 nodal stage, extranodal extension (ENE), nodal classification	Extranodal extension (ENE)	Disease-specific survival (DSS); prognostic performance of AJCC8 nodal staging	AJCC8 upstaged a large proportion of patients because of ENE but showed only modest prognostic discrimination. Disease-specific survival did not differ significantly between several AJCC8 nodal categories, indicating limited predictive accuracy.	The AJCC8 nodal staging system has limited prognostic value for metastatic head and neck cutaneous SCC and requires further refinement to improve risk stratification.	Retrospective study limited to patients with nodal metastatic disease; findings may not be generalizable to all patients with localized cSCC. No direct reconstructive, functional, complication, or aesthetic endpoint was attributed unless explicitly reported.

No.	Study ID (Author, Year)	Study Design	N	Clinical Predictor	Histopathological Predictor	Outcome	Reported study-level findings	Oncologic interpretation	Limitations / reconstructive relevance
**16**	Ran et al., 2025	Multicenter retrospective cohort study	16844 tumors	Tumor diameter ≥2 cm; number of risk factors	Poor differentiation, tumor extension beyond subcutaneous fat, large-caliber perineural invasion	Local recurrence, nodal metastasis, distant metastasis, disease-specific death	Increasing numbers of high-risk features were associated with progressively higher rates of local recurrence, nodal metastasis, distant metastasis, and disease-specific death. Tumors with three risk factors had substantially worse outcomes than those with two risk factors, despite both being classified as BWH T2b.	The cumulative number of clinicopathological risk factors provides meaningful prognostic information and may improve risk stratification beyond the current BWH staging system.	Retrospective multicenter design; patients with metastatic disease at presentation or adjuvant therapy were excluded, which may limit generalizability to all cSCC populations. No direct reconstructive, functional, complication, or aesthetic endpoint was attributed unless explicitly reported.
**17**	de Jong et al.; 2024	Prospective multicenter cohort study	514 solid organ transplant recipients (623 primary cSCCs)	Head and neck location, local recurrence, tumor size >2 cm, clinical ulceration	Poor differentiation, perineural invasion, deep invasion, high tumor stage	Metastasis (2-year cumulative incidence)	Head and neck location, local recurrence, tumor size >2 cm, ulceration, poor differentiation, perineural invasion, and deep invasion were independent predictors of metastasis. High-stage ulcerated tumors had the highest metastatic risk, with a 2-year cumulative incidence of 46.2%.	Solid organ transplant recipients have a markedly increased risk of metastatic cSCC, and established clinicopathological factors remain reliable predictors of disease progression and metastasis.	Restricted to solid organ transplant recipients, limiting generalizability to immunocompetent patients; minimum follow-up of 2 years may underestimate late metastatic events. No direct reconstructive, functional, complication, or aesthetic endpoint was attributed unless explicitly reported.

No.	Study ID (Author, Year)	Study Design	N	Clinical Predictor	Histopathological Predictor	Outcome	Reported study-level findings	Oncologic interpretation	Limitations / reconstructive relevance
**18**	Shahwan et al.; 2025	Multicenter retrospective cohort study	15481 BWH T1 cutaneous squamous cell carcinomas	Tumor diameter 1–2 cm; number of minor risk factors	Moderate differentiation, subcutaneous fat invasion, small-caliber perineural invasion	Local recurrence, metastasis, major poor outcomes, disease-specific death	Moderate differentiation, tumor diameter 1–2 cm, and subcutaneous fat invasion were independently associated with poor outcomes. The risk of metastasis and major poor outcomes increased progressively with the accumulation of minor risk factors, exceeding 5% in tumors with three minor risk factors.	Multiple minor risk factors significantly worsen prognosis even in BWH T1 tumors, supporting closer surveillance and consideration of their incorporation into future staging systems	Retrospective multicenter design; relatively small number of major poor outcome events despite the large cohort. No direct reconstructive, functional, complication, or aesthetic endpoint was attributed unless explicitly reported.
**19**	Ebrahimi et al.; 2020	Multicenter retrospective cohort study	1146 patients with nodal metastatic HNcSCC	Number and size of nodal metastases, immunosuppression	Perineural invasion	Disease-specific survival (DSS)	Significant prognostic heterogeneity was observed within AJCC pN2a, pN3b, and stage IV categories. The number and size of nodal metastases, immunosuppression, and perineural invasion independently influenced survival, with 5-year DSS ranging from 90% to 60% among stage IV subgroups.	The AJCC staging system inadequately stratifies prognosis in metastatic HNcSCC because of substantial heterogeneity within advanced nodal and stage IV categories.	Retrospective design; included only patients with nodal metastatic disease, limiting applicability to patients with localized cSCC. No direct reconstructive, functional, complication, or aesthetic endpoint was attributed unless explicitly reported.

No.	Study ID (Author, Year)	Study Design	N	Clinical Predictor	Histopathological Predictor	Outcome	Reported study-level findings	Oncologic interpretation	Limitations / reconstructive relevance
**20**	Costantino et al.; 2025	Systematic review and meta-analysis	2076 patients with metastatic cutaneous head and neck squamous cell carcinoma	Extranodal extension (ENE)	Extranodal extension (ENE)	Overall survival (OS), disease-free survival (DFS)	ENE was present in 65.6% of patients but was not significantly associated with worse overall or disease-free survival in the pooled analysis, suggesting limited independent prognostic value.	ENE alone may not be a reliable prognostic factor in metastatic cutaneous head and neck squamous cell carcinoma, highlighting the need for refined risk stratification and improvements to current nodal staging systems.	Limited by the retrospective nature and heterogeneity of the included studies; prospective validation is required. No direct reconstructive, functional, complication, or aesthetic endpoint was attributed unless explicitly reported.
**21**	Hirotsu et al.; 2025	Multicenter retrospective cohort study	23166 cutaneous squamous cell carcinomas	BWH stage, clinicopathologic characteristics	Lymphovascular invasion (LVI)	Major poor outcomes (nodal metastasis, in-transit metastasis, distant metastasis, disease-specific death)	LVI-positive tumors had significantly higher rates of major poor outcomes than LVI-negative tumors. LVI remained an independent predictor after adjustment and identified high-risk patients even among low-stage (BWH T1–T2a) tumors.	Lymphovascular invasion is an independent prognostic factor for metastasis and disease-specific death and should be considered in future risk stratification models and staging systems for cSCC.	Retrospective design; low prevalence of LVI-positive tumors (0.8%), which may limit precision despite the large cohort. No direct reconstructive, functional, complication, or aesthetic endpoint was attributed unless explicitly reported.
**22**	Ebrahimi et al.; 2021	Multicenter retrospective cohort study	990 patients with metastatic head and neck cutaneous squamous cell carcinoma	Number and size of nodal metastases, extranodal extension, nodal stage	Extranodal extension (ENE)	Disease-specific survival (DSS), overall survival (OS), prognostic performance of AJCC8, N1S3, and ITEM staging systems	AJCC8 classified most patients as stage IV because of extranodal extension, resulting in poor discrimination between prognostic groups. N1S3 and ITEM provided better patient distribution and modestly improved prognostic performance, although all three systems showed limited predictive accuracy.	Current AJCC8 nodal staging has limited prognostic utility in metastatic HNcSCC. More accurate, disease-specific staging systems are needed to improve risk stratification.	Retrospective design; study limited to patients with nodal metastatic HNcSCC treated with curative intent; predictive performance of all evaluated staging systems remained only moderate. No direct reconstructive, functional, complication, or aesthetic endpoint was attributed unless explicitly reported.

No.	Study ID (Author, Year)	Study Design	N	Clinical Predictor	Histopathological Predictor	Outcome	Reported study-level findings	Oncologic interpretation	Limitations / reconstructive relevance
**23**	Fermi et al.; 2025	Multicenter retrospective observational study	68 patients with nodal metastatic head and neck cutaneous squamous cell carcinoma	Immunosuppression, sex, nodal dissemination pattern, adjuvant radiotherapy	Perineural invasion (PNI), extranodal extension (ENE), advanced nodal stage	Disease-free survival (DFS), disease-specific survival (DSS), overall survival (OS)	ENE in parotid and cervical lymph node metastases and PNI were independent predictors of poorer survival. Advanced nodal disease and omission of adjuvant radiotherapy were associated with worse outcomes. Immunosuppression influenced the pattern of nodal dissemination but was not independently associated with survival.	Perineural invasion, extranodal extension, advanced nodal stage, and lack of adjuvant radiotherapy are major prognostic factors in metastatic head and neck cSCC, supporting their use in postoperative risk stratification.	Retrospective design; relatively small sample size; findings limited to patients with nodal metastatic head and neck cSCC treated at three Italian centers. No direct reconstructive, functional, complication, or aesthetic endpoint was attributed unless explicitly reported.
**24**	Ebrahimi et al.; 2020	Multicenter retrospective cohort study	1128 patients with nodal metastatic head and neck cutaneous squamous cell carcinoma	Number of nodal metastases, AJCC N stage, TNM stage	Nodal metastatic burden	Disease-specific survival (DSS), overall survival (OS)	The number of nodal metastases was an independent predictor of mortality. Patients with 3–4 and ≥5 metastatic lymph nodes had progressively worse disease-specific and overall survival compared with those with 1–2 involved nodes. The proposed nodal categories (1–2, 3–4, ≥5 nodes) provided superior prognostic discrimination compared with the AJCC TNM staging system.	The number of metastatic lymph nodes is a powerful independent prognostic factor and may improve future staging systems and risk stratification in head and neck cSCC.	Retrospective design; included only patients with nodal metastatic HNcSCC treated with curative intent; external validation of the proposed nodal categories is required. No direct reconstructive, functional, complication, or aesthetic endpoint was attributed unless explicitly reported.

No.	Study ID (Author, Year)	Study Design	N	Clinical Predictor	Histopathological Predictor	Outcome	Reported study-level findings	Oncologic interpretation	Limitations / reconstructive relevance
**25**	Manyam et al.; 2017	Multicenter retrospective cohort study	205 patients with head and neck cutaneous squamous cell carcinoma	Immunosuppression, recurrent disease	Poor differentiation, perineural invasion (PNI)	Overall survival (OS), locoregional recurrence-free survival (LRRFS), progression-free survival (PFS)	Immunosuppressed patients had significantly worse locoregional recurrence-free survival and progression-free survival than immunocompetent patients despite surgery and postoperative radiotherapy. On multivariable analysis, immunosuppression, recurrent disease, poor differentiation, and perineural invasion were independent predictors of locoregional recurrence.	Immunosuppression is a major independent adverse prognostic factor in head and neck cSCC and should be incorporated into prognostic models, treatment strategies, and clinical trial design.	Retrospective design; included only patients treated with surgery and postoperative radiotherapy, which may limit generalizability to all cSCC populations. No direct reconstructive, functional, complication, or aesthetic endpoint was attributed unless explicitly reported.
**26**	Ebrahimi et al.; 2024	Multicenter retrospective cohort study	1151 patients	Immunosuppression; bilateral nodal disease; nodal size >6 cm; ≥5 nodal metastases	None independently identified (focus on nodal disease characteristics)	Distant metastatic recurrence (DMR)	The 5-year risk of distant metastatic recurrence was 9.6%. Immunosuppression, nodal size >6 cm, ≥5 metastatic lymph nodes, and bilateral nodal disease independently predicted DMR. Based on these variables, the authors developed a simple DMR risk score that stratified patients from very low to extremely high risk of distant recurrence.	Clinical characteristics of nodal metastatic disease can accurately identify patients at increased risk of distant metastasis and may support individualized treatment planning and surveillance.	Retrospective design; included only patients with nodal metastatic HNcSCC treated with curative intent; results may not be generalizable to all cSCC patients or non-head-and-neck tumors. No direct reconstructive, functional, complication, or aesthetic endpoint was attributed unless explicitly reported.

No.	Study ID (Author, Year)	Study Design	N	Clinical Predictor	Histopathological Predictor	Outcome	Reported study-level findings	Oncologic interpretation	Limitations / reconstructive relevance
**27**	Girardi et al.; 2025	Multicenter retrospective cohort study	3168 patients (9,852 tumors)	AJCC8 and BWH T stage; tumor stage according to established staging systems	Histopathological features incorporated within the BWH and AJCC8 staging criteria (e.g., tumor diameter, depth of invasion, differentiation, perineural invasion)	Local recurrence, nodal recurrence, distant recurrence, disease-specific death	Both AJCC8 and BWH staging systems demonstrated similar performance in predicting disease-specific death, with moderate prognostic discrimination. The BWH system showed better separation for local recurrence, whereas both systems performed less effectively for nodal and distant recurrence, with high specificity but low sensitivity.	Current AJCC8 and BWH staging systems are useful for predicting survival in head and neck cSCC, but additional prognostic variables are needed to improve prediction of recurrence and metastasis in future staging models.	Retrospective design; only surgically treated patients with curative intent were included; exclusion of recurrent tumors and patients receiving prior head and neck treatment may limit generalizability. No direct reconstructive, functional, complication, or aesthetic endpoint was attributed unless explicitly reported.
**28**	Mooney et al.; 2020	Multicenter prospective cohort study	105 high-risk tumors	Presence of ≥4 high-risk features; tumor depth >5 mm	Perineural invasion (PNI); depth of invasion	Sentinel lymph node positivity, regional nodal metastasis, disease-specific survival	Sentinel lymph node biopsy identified occult nodal metastases in 9.5% of patients, with an overall subclinical nodal metastasis rate of 14.3%. The presence of four or more high-risk features or the combination of tumor depth >5 mm and PNI significantly predicted nodal metastasis. Patients with nodal metastases had significantly worse disease-specific survival.	Sentinel lymph node biopsy is a feasible staging tool for high-risk head and neck cSCC, and combinations of high-risk clinicopathological features predict nodal metastasis more accurately than individual risk factors alone.	Moderate sample size; limited to clinically node-negative high-risk head and neck cSCC; sentinel node biopsy is not standard practice in all centers, which may limit generalizability. No direct reconstructive, functional, complication, or aesthetic endpoint was attributed unless explicitly reported.

No.	Study ID (Author, Year)	Study Design	N	Clinical Predictor	Histopathological Predictor	Outcome	Reported study-level findings	Oncologic interpretation	Limitations / reconstructive relevance
**29**	Campoli et al.; 2014	Multicenter prospective cohort study	753 patients	Head and neck location, larger tumor diameter, clinically palpable lymphadenopathy, recurrent tumors, pain	Perineural invasion (PNI), poor differentiation, greater tumor thickness	Association of incidental PNI with adverse clinicopathological features	Incidental PNI was identified in 4.6% of tumors and was significantly associated with head and neck location, larger tumor size, recurrent disease, palpable lymphadenopathy, poor differentiation, and increased tumor thickness, all recognized indicators of poor prognosis.	Incidental perineural invasion is strongly associated with multiple adverse clinicopathological features and may serve as an important marker for improved prognostic assessment in cutaneous squamous cell carcinoma.	Relatively low number of PNI-positive cases; study evaluated associations with adverse prognostic factors rather than long-term survival outcomes. No direct reconstructive, functional, complication, or aesthetic endpoint was attributed unless explicitly reported.

Supplementary Table S2. Distinct contextual reconstructive, functional, aesthetic, technical, and methodological sources used in the narrative framework (n = 13)

The 29 prognostic publications charted in Table S1 are not repeated here. No reconstructive technique or aesthetic consequence was attributed to a prognostic publication unless that endpoint was explicitly measured. The 13 sources below are contextual and were identified through reference-list screening; they do not constitute an exhaustive systematic reconstructive dataset.

No.	Source	Design / population	Site	Endpoints actually measured or addressed	Verified source-level finding	Evidence category	Applicability / limitation
1	Mureau et al., 2007	Clinical outcome study; 38 consecutive patients with subtotal nasal defects; mixed malignancies	Nose	Standardized interviews, examination, and photographs; functional and aesthetic outcomes	Objective irregularities were common, while 79% of patients were very satisfied with total nasal appearance.	Direct aesthetic/functional evidence	Not HNcSCC-specific; nasal reconstruction only.
2	Moolenburgh et al., 2008 (patient vs panel)	Clinical aesthetic follow-up; 39 patients, 33 assessed	Nose	Patient and professional-panel ratings using standardized photographs	Patients rated total appearance more favorably than the professional panel (4.2 vs 3.5; $p = 0.03$).	Direct aesthetic evidence	Mixed histology; site-specific; subjective ratings.
3	Moolenburgh et al., 2008 (lay observers)	Observer-perception study	Nose/face	Layperson ratings of attractiveness and facial abnormality	The study measured a lay-observer perspective distinct from patient or professional-panel assessment.	Direct observer-rated aesthetic evidence	Site-specific; mixed histology; does not measure HNcSCC prognosis.
4	Menick, 2010	Technical review	Nose	Anatomical analysis; lining, structural support, cover, and staged reconstruction	Major nasal defects may require staged replacement of lining, support, and cover; this is technical guidance rather than comparative prediction.	Technical/conceptual source	Not a comparative cohort; no HNcSCC-specific predictive analysis.
5	Menick, 2009	Technical review	Nose	Paramedian forehead-flap planning and staged reconstruction	Describes indications and staged technical principles for forehead-flap nasal reconstruction.	Technical/conceptual source	Not a comparative HNcSCC study.
6	Lin et al., 2008	Technical review	Scalp and calvarium	Defect analysis and reconstructive options	Technique selection is described according to defect size, location, depth, exposed structures, and tissue requirements.	Technical/conceptual source	Review based on mixed etiologies; no HNcSCC-specific prediction.
7	Leedy et al., 2005	Algorithmic technical review	Scalp	Primary closure, grafts, local/regional flaps, and free tissue transfer	Presents an algorithmic approach based on acquired scalp-defect characteristics.	Technical/conceptual source	Mixed etiologies; technique guidance rather than comparative outcome evidence.
8	Seitz and Gottlieb, 2009	Technical review	Scalp and forehead	Defect size, location, depth, tissue quality, exposed structures, and reconstructive goals	Emphasizes analysis of the anticipated defect and surrounding tissue quality when selecting reconstruction.	Technical/conceptual source	Mixed etiologies; no HNcSCC-specific predictive model.
9	van Driel et al., 2010	Retrospective two-centre cohort; 84 patients with malignant scalp/forehead defects	Scalp and forehead	Free-flap outcomes, flap failure, aesthetic ratings, revisions, survival	Mean defect area was 134 cm^2; total flap failure was 6%; aesthetic revision was performed in 23%; frontal location received lower panel ratings ($p = 0.004$).	Direct reconstructive/aesthetic evidence	Mixed malignancies; selected complex microsurgical cases.
10	Iblher et al., 2010	Retrospective comparative series; 60 scalp-tumor patients	Scalp	Tumor/defect characteristics, reconstruction, complications, function, aesthetics, recurrence, and survival	Five reconstructive categories were used to develop an oncologic scalp-reconstruction algorithm.	Direct reconstructive evidence	Retrospective; mixed tumor types; algorithm requires external validation.
11	Chao et al., 2012	Retrospective microsurgical cohort; 138 reconstructions in patients with cancer	Scalp and calvarium	Perioperative and late complications; scalp-only vs simultaneous calvarial reconstruction	Perioperative complications occurred in 21.0% and late recipient-site complications in 11.6%; rates did not differ significantly between comparison groups.	Direct reconstructive/complication evidence	Cancer population but not HNcSCC-specific; free-flap selection bias.
12	Stewart et al., 2004	Instrument development and validation	Nasal airway	Patient-reported nasal obstruction (NOSE scale)	Developed and validated a disease-specific patient-reported measure of nasal obstruction.	Outcome-measurement methodology	Not designed specifically for oncologic reconstruction.

No.	Source	Design / population	Site	Endpoints actually measured or addressed	Verified source-level finding	Evidence category	Applicability / limitation
13	Draaijers et al., 2004	Instrument development and validation	Scar assessment	Patient and observer scar assessment (POSAS)	Developed a feasible parallel patient/observer scar-assessment instrument.	Outcome-measurement methodology	Initial validation was not specific to HNcSCC or facial reconstruction.
